# Application of Loop-Mediated Isothermal Amplification (LAMP) in Sex Identification of Parrots Bred in Egypt

**DOI:** 10.3390/biology11040565

**Published:** 2022-04-08

**Authors:** Sara M. Elnomrosy, Naglaa M. Hagag, Mohamed I. AbdAllah, Rafał Kolenda, Maciej Zacharski

**Affiliations:** 1 Genome Research Unit, Animal Health Research Institute (AHRI), Agriculture Research Center (ARC), Nadi El-Said Street, Dokki, Giza 12618, Egypt; dr_sara_vet_2007@yahoo.com (S.M.E.); naglaahagagahri@gmail.com (N.M.H.); yja_mohamed75@yahoo.com (M.I.A.); 2 Department of Biochemistry and Molecular Biology, Faculty of Veterinary Medicine, Wroclaw University of Environmental and Life Sciences, 50-375 Wroclaw, Poland; rafal.kolenda@upwr.edu.pl

**Keywords:** parrots, loop-mediated isothermal amplification, LAMP, PCR, sex determination, noninvasive

## Abstract

**Simple Summary:**

Loop-mediated isothermal amplification is an enzymatic reaction that allows for specific amplification of a chosen DNA fragment. It works at a constant temperature without the need for expensive equipment. The reaction result can be verified without electrophoresis making it ideal for field application or poorly resourced laboratories. In this work, the authors have compared the LAMP and PCR performance in molecular sex identification of parrots using the DNA extracted from the feathers. The authors examined samples from 21 parrot species. They show that PSI-W primers and LAMP are suitable for sexing 17 species not tested previously.

**Abstract:**

Over 400 of the 3800 tropical avian species are endangered or threatened. One of many solutions to conserve animal biodiversity is breeding animals in zoos or private animal farms. Animal breeding programs are difficult to implement in species with sexual monomorphism, such as parrots. Molecular biology methods offer a solution to determine the sex of these species. Therefore, in this study, we aimed to test the performance of PCR and LAMP techniques on sex identification for 21 parrot species belonging to three families, i.e., *Psittacidae*, *Cacatuidae*, and *Psittaculidae*. We established a protocol for DNA isolation from feathers in our laboratory and found optimal conditions for PCR and LAMP. We showed that the LAMP method with the use of the PSI-W primers set, developed by Centeno-Cuadros, functions in 17 previously untested species. Moreover, we found that further improvements are required in universal LAMP primers for the detection of parrot DNA, which are necessary for confirmation of the male sex. The LAMP method also proved to be more sensitive for female sex identification in contrast to the reference PCR test. Therefore, we conclude that LAMP is a suitable method for the routine diagnostic sex identification of parrots.

## 1. Introduction

More than 400 of the 3800 tropical avian species are endangered or threatened [1]. According to The International Union for Conservation of Nature (IUCN2019), 16 parrot species are extinct, 16 are critically endangered, 36 are endangered, 56 are vulnerable, and 57 are near threatened. The reduction in wild parrot populations results from hunting for food or trade, habitat loss, and competition from invasive species [2,3]. Therefore, the preservation of these various species from extinction is urgent and intensive bird breeding programs might help in some instances, e.g., to reduce the number of birds captured from the wild for pets.

Approximately 50% of bird species are monomorphic [4], which makes sex determination by their external morphology hardly possible for nestlings, and problematic even after puberty. These difficulties cause the breeding of monomorphic birds to be labour intensive and time-consuming. The high demand for the parrots and lack of captive breeding success may encourage the illegal import of young birds [5].

A variety of methods have been developed over the last decade to identify the sex of birds. These methods include behavioural assays, examinations of sex organs, hormone analysis, etc. Although many methods exist, these approaches range in accuracy and can be laborious, expensive, and invasive [6]. The relatively recent advancement in molecular techniques has improved our ability to perform sexing with the use of the DNA isolated from non-invasive tissue samples, such as feathers [7]. Minimizing the birds’ stress by reducing bird handling time and the use of non-invasive methods is critical during sampling of endangered species, chicks, and fragile birds [8]. Therefore, using feathers and scats as the DNA source seems to be the best solution [9,10]. The first successful bird sex determination by the detection of the Chromo Helicase DNA-binding gene (*chd-1*) located on the W chromosome using PCR was carried out on DNA isolated from the feathers of the Spix’s macaw (*Cyanopsitta spixii*) [11]. This gene serves as a molecular marker for sex identification in birds due to its high degree of conservation and differences in length between Z and W chromosomes, caused by diverse intron sizes [12].

Fridolfsson et al. designed 2550F and 2718R primers to amplify the *chdw* and *chdz* genes allowing the accurate identification of sex in several avian species. When used with DNA extracted from heterogametic birds, they produced two bands with an expected size differences of 150–250 bp, which permits Z- and W- band visualization on an agarose gel [13]. These primers facilitated the successful sex determination of several parrot species, e.g., *Psittacus erithacus*, *Amazona leucocephala*, *Aratinga solstitialis*, *Pyrrhura molinae*, *Psittacula eupatria*, *Ara ararauna*, *Ara militaris*, *Myiopsitta monachus* and *Psittacula krameri*. However, in some species, they required optimization of PCR conditions such as annealing temperature, PCR product reamplification or use of touchdown PCR [8,10,14,15,16].

Although the introduction of PCR as a method to identify sex decreased the stress inflicted on the animals, this approach still has some major drawbacks. The method requires expensive reagents and specialized laboratories equipped with advanced machines such as thermocycler and gel electrophoresis apparatus. Moreover, the test is time and labour-consuming and, including sample transportation, may need several days to deliver the results [17]. Loop-Mediated Isothermal Amplification (LAMP) overcomes most of these shortcomings with uniform temperature for sample incubation and easy end-point result detection. Therefore, it is considered to be an effective diagnostic tool for developing countries as there is no need for costly equipment and skilled staff [18].

The LAMP technique, developed and described in detail by Notomi et al., uses the *Bst* DNA polymerase with high strand displacement activity that facilitates auto-cycling and DNA synthesis [19]. It requires at least two pairs of highly specific primers [20]. The first primer pair named forward outer (F3) and backward outer (B3) enables DNA strand displacement. The second pair of primers, i.e., forward inner primer (FIP) and backward inner primer (BIP), allow loop formation. The amplified DNA can be detected by several methods, e.g., gel electrophoresis, measurement of fluorescence after addition of SYBR Green or other DNA binding fluorescent dyes [18], naked eye turbidity observation [21], a colorimetric method with the use of metal indicator [22] or colorimetric method with the use of pH indicator [23].

The first successful attempt to use LAMP for sex identification in wild birds was based on the detection of *chd* gene conducted on the three raptor species belonging to the *Accipitridae* family, i.e., *Gyps Fulvus*, *Milvus migrans*, and *Neophron percnopterus* using the ACCIW and ACCIZ primer sets [17]. Since then, several bird family specific primer sets have been developed and tested across different avian species (*Psittacidae* (parrots): PSI-W; *Estrildidae*: EST-W; *Icteridae*: ICT-W, ICT-Z; *Ciconiidae*: CIC-W, CIC-Z) [24]. The development of NEO-W primers enabled sex detection of species belonging to 12 orders of *Neognathae* superorder [24]. UCE primer set targeting ultra-conserved elements on the autosomal chromosome 6 served as a positive control of DNA amplification to discriminate between false negatives and actual males [17]. Even with these NEO-W universal primers, it is essential to optimize the LAMP incubation time and temperature separately for each species [24]. Unfortunately, this method was not suitable for sex determination of parrots, one-third of which are threatened by extinction (IUCN2019). Adapting the method for the *Psittacidae* family required a specific primer set—PSI-W [24]. As reported by Ceteno-Cuados, LAMP, contrary to PCR enables in situ molecular sex identification in birds, facilitating the work of ornithologists, ecologists, researchers as well as commercial and private breeders. The cost comparison of both methods is complicated and depends on several factors [24]. We can say, that establishing this method is justifiable from the economic point of view if the laboratory is not equipped with the thermocycler.

In this study, we validated the LAMP method developed by Centeno-Cuadros for the molecular sex identification in birds of 21 species, bred and reproduced in Egypt, belonging to three different families, i.e., *Psittacidae*, *Cacatuidae*, and *Psittaculidae*. The method has not been previously tested in 17 of 21 species studied. Additionally, we reported an optimized protocol for DNA extraction from non-invasive feather samples. We compared the results obtained from the LAMP and PCR-based method.

## 2. Material and Methods

### 2.1. Feather Samples

A total of 150 parrots from 21 species were sampled for sex identification (Table 1). The feather samples were provided by private breeders and Egyptian zoos. Between 3 to 10 throat feathers were collected by plucking. Each sample was stored in a zipper bag and labelled. Sampling was carried in accordance with the Ornithological Council’s guidelines on the use of wild birds in research [25]. The samples were shipped to the laboratory at room temperature within 1 week from collection or stored at +4 °C upon shipment.

### 2.2. DNA Isolation

DNA was extracted from samples within 2 weeks from shipment to the Egyptian laboratory. The quills from 3 to 10 throat feathers were cut with a scalpel blade into 2–5 mm fragments and transferred into sterile 1.5 mL Eppendorf tubes. For each sample, a new blade was used. DNA was isolated with a Genomic Mini Kit according to the manufacturer’s instructions with slight modifications (A&A Biotechnology, Gdańsk, Poland). Briefly, 100 µL of TRIS buffer, 50 µL of lysis buffer and 10 µL of 1M DTT (freshly prepared) were added to fragmented quills, vortexed and incubated at 50 °C overnight. Next, samples were placed at 70 °C for 5 min to inactivate proteinase K, followed by 20 s of vortexing and centrifugation at maximum speed. Subsequently, the lysate was transferred onto the minicolumns supplied by the manufacturer and the tubes were centrifuged. DNA bound to the resin was washed twice with A1 buffer. In the end, DNA was eluted with 50 µL of TRIS elution buffer (preheated to 70 °C). The purified DNA was stored at −20 °C. The concentrations of extracted DNA were measured with SPECTRO star^Nano^ (BMG LABTECH, Ortenberg, Germany).

### 2.3. Sex Identification with PCR

The Chromo Helicase DNA-binding genes (*chdw*/*chdz*) were amplified by PCR targeting the *chd1* intron region, according to Fridolfsson and Ellegren [13] with minor modifications. PCR was carried out with primers 2550F and 2718R synthesized at Sigma (Welwyn Garden City, UK) (Table 2). The PCR mix contained double distilled water, DreamTaq Green buffer (10×), 0.2 mM dNTPs, 1 U of DreamTaq polymerase (Thermo, Cat. no. EP0713, Waltham, MA, USA), 0.2 µM of each primer and 15–25 ng of DNA template or water for no template control (NTC). PCR was performed with an initial denaturation at 95 °C for 3 min, followed by 40 cycles of denaturation (95 °C for 30 s), annealing (50–56 °C for 30 s, differed according to species) and extension (72 °C for 1 min). The optimal annealing temperature for each species was determined with the use of gradient PCR [12]. The temperature gradient of annealing was tested in the range from 50 °C to 60 °C every 2 °C.

### 2.4. Optimization of LAMP for Sex Identification

First, LAMP reactions were optimized using the DNA template of one female to adjust the optimal temperature and incubation time for each species. DNA used for the LAMP was diluted to 20–25 ng/µL. Each LAMP reaction was performed in a volume of 25 µL and contained: water, 12.5 µL WarmStart LAMP 2X Master Mix (New England Biolabs, Ipswich, MA, USA), 2.5 µL LAMP primers mix (Table 2), 15–25 ng of DNA or water for NTC. Reactions were incubated in following temperatures: 57 °C, 59 °C, 61 °C, 63 °C and 65 °C for 30, 45, 60 or 80 min.

### 2.5. PCR and LAMP Amplification Detection

All PCR and LAMP products were analysed with agarose gel electrophoresis. First, agarose was melted in the TRIS-Borate-EDTA buffer and 1.5–2.5% gels with the addition of Midori Green Stain were cast. DNA fragments were resolved during the electrophoresis in the agarose gels and visualized with UV light and FireReader V10 (UVITEC, Cambridge, UK).

### 2.6. Figures and Statistical Analysis

Figures were generated with ggplot2 implemented in R software [26,27]. Statistical comparisons of PCR and LAMP methods were performed with McNemar’s test [28].

## 3. Results

### 3.1. Sample Collection and DNA Isolation

Overall, 150 samples from 21 parrot species were collected from private breeders and Zoos in Egypt. According to The International Union for Conservation of Nature (IUCN) Red List of Threatened Species, five DNA samples originated from critically endangered parrot species. Moreover, thirteen and two samples originated from endangered and vulnerable species, respectively. The rest of the specimens originated from near threatened and least-concern parrot species. DNA isolation protocol from feather quills was established in our laboratory and successfully applied to all the specimens. As a result, the concentration of isolated DNA ranged from 4.9 to 133 ng/µL.

### 3.2. Parrot Sex Identification with PCR

First, optimal annealing temperatures for primers 2550F and 2718R were determined (Table 3). Next, DNA from 150 parrots was used in PCR to determine the sex of the birds. The sex was detected for 93.3% of specimens, with 52.0% and 41.3% parrots identified as female and male, respectively. For each of the 21 parrot species analysed, at least one female and one male were found. Ten samples used in PCR did not result in successful amplification. These samples came from four parrot species: *Nymphicus hollandicus* (5), *Amazona aestiva* (3), *Amazona farinosa* (1), and *Lorius lory* (1). For the samples from *Amazona aestiva* and *Lorius lory*, the mean DNA concentration of PCR negative samples (32 ng/μL and 9 ng/μL) was lower than the PCR-positive (88 ng/μL and 28 ng/μL, respectively). On the other side, this was not true for *Amazona farinose*, *Nymphicus hollandicus*, where PCR negative samples had higher DNA concentration (24 ng/μL and 62 ng/μL) than the PCR-positive ones (10 ng/μL and 17 ng/μL).

### 3.3. Parrot Sex Identification with LAMP

**Figure 1:**
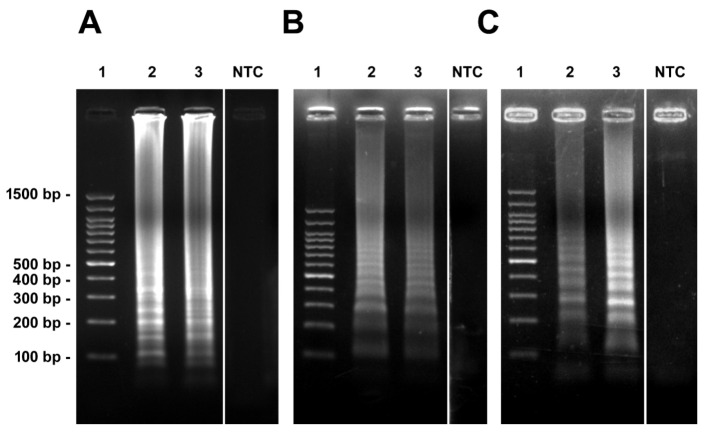
Representative results of gel electrophoresis of LAMP products amplified with the use of PSI-W primers (**A**), UCE primers (**B**), and CIC-Z primers (**C**). Amplification products were separated on 2.5% agarose gel. (**A**) Lane 1—100 bp DNA Ladder (Transgen Biotech, Beijing, China), Lane 2—*Amazona aestiva*, Lane 3—*Amazona farinosa*; (**B**) Lane 1—100 bp DNA Ladder (Transgen Biotech), Lane 2—*Amazona amazonica*, Lane 3—*Psittacula krameri*; (**C**) Lane 1—100 bp DNA Ladder (Transgen Biotech), Lane 2—*Poicephalus senegalus*, Lane 3—*Poicephalus meyeri*. Each panel includes NTC lane which is a representative image of No Template Control LAMP reaction with specific primers.

First, DNA from one female or male (sex determined by PCR) for each parrot species investigated in this study was used to find optimal LAMP reaction temperatures and time for primer sets PSI-W, CIC-Z, and UCE. The optimal incubation time for all primer sets was found to be one hour. Overall, only PSI-W gave results in all parrot species included in this study (Figure 1A) and optimal reaction temperatures ranged from 57 °C to 63 °C (Table 3). The use of the UCE primer set resulted in DNA amplification only in five species and the optimal temperature of the reaction was 57 °C (Figure 1B). CIC-Z primers also worked only in one temperature, but in this case, it was 63 °C and nucleic acid amplification was detected in 10 parrot species (Figure 1C).

**Figure 2:**
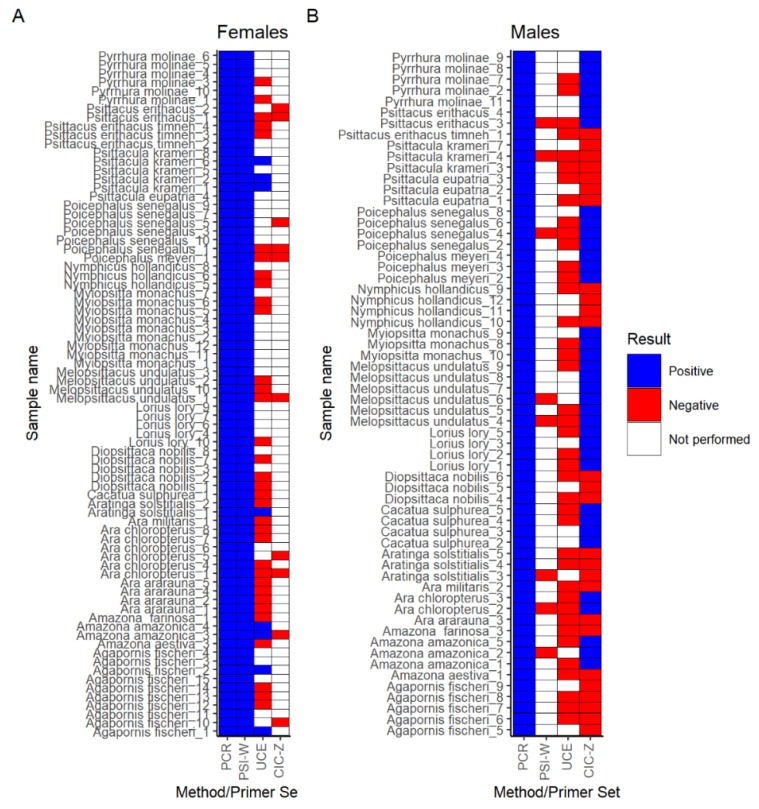
Results for LAMP amplification with parrot DNA for (**A**) females and (**B**) males. DNA samples positive in PCR were subjected to LAMP analysis with primer sets PSI-W, UCE, and CIC-Z. Parrot species and samples numbers are shown on the y-axis. Names of method (PCR) or primer set (PSI-W, UCE, CIC-Z) are shown on the x-axis. Each rectangle shows result of amplification, and the colours correspond to the reaction outcome: blue—positive, red—negative, white—reaction was not performed for this sample.

Optimized LAMP reactions for the aforementioned three primer sets were employed in LAMP on DNA samples that gave positive (140) and negative (10) results in PCR. LAMP with PSI-W primer set worked with all 78 feather specimens originating from females (Figure 2A). PSI-W primer set was also tested on eight male DNA samples, and they exhibited negative results (Figure 2B). LAMP reactions with UCE primers were tested on 82 DNA samples, but only eight (all females) resulted in DNA amplification (Figure 2). Subsequently, 78 DNA samples were used to test CIC-Z primers and gave positive results for 36 samples (all males) (Figure 2).

### 3.4. Comparison of PCR and LAMP Techniques for Sex Identification in Parrots

**Figure 3:**
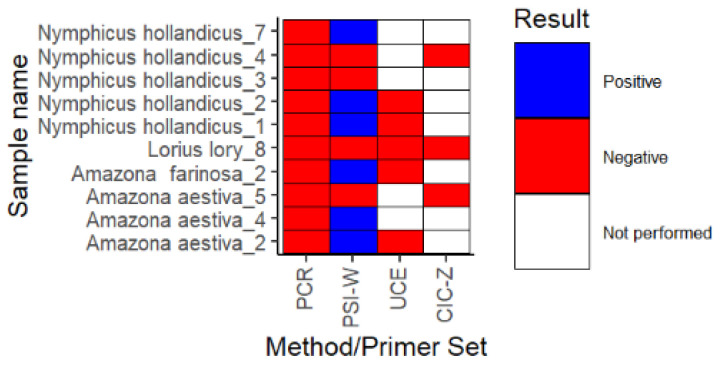
Results for LAMP amplification in samples with negative PCR results. DNA samples negative in PCR were subjected to LAMP analysis with primer sets PSI-W, UCE, and CIC-Z. Parrot species and samples numbers are shown on the y-axis. Names of method (PCR) or primer set (PSI-W, UCE, CIC-Z) are shown on the x-axis. Each rectangle shows results of amplification and the colours correspond to the reaction outcome: blue—positive, red—negative, white—reaction was not performed for this sample.

PCR used in this study failed to determine female sex in seven samples that gave positive results by LAMP with PSI-W primer sets (Figure 3). These samples belonged to birds from three species: *Amazona aestiva* and *farinosa*, and *Nymphicus hollandicus*. On the other hand, all the DNA samples from parrots determined as female by PCR gave identical results in the LAMP. Therefore, the latter method outperformed PCR in female sex identification (*p* < 0.001, McNemar’s test). Confirmation of male sex in LAMP (negative result with PSI-W and positive results with UCE or CIC-Z) was successful for five species: *Ara chloropterus*, *Amazona amazonica*, *Poicephalus senegalus*, *Psittacus erithacus*, *Melopsittacus undulatus*. The use of PCR was more sensitive than LAMP in the verification of male sex (*p* < 0.001, McNemar’s test). Both methods did not determine the sex of three parrots and these samples originated from three different species, i.e., *Amazona aestiva*, *Nymphicus hollandicus*, and *Lorius lory* (Figure 3).

## 4. Discussion

Human activities in the last century have led to irreversible changes in the earth’s ecosystems. Although multiple actions promoting biodiversity preservation have been implemented, still up to one million animal and plant species face extinction in the coming years [29]. One of the solutions contributing to animal biodiversity conservation is breeding animals in zoos or private animal farms [30]. Animal breeding programs are difficult to implement in species with sexual monomorphism. Molecular biology methods offer a solution to determine the sex in these species [31,32]. This also applies to many parrot species, where sexual monomorphism leads to serious problems in reproduction [33]. In this study, we aimed to test the performance of PCR and LAMP techniques as tools to identify the sex of parrot species bred in Egypt. First, we gathered a representative collection of feather samples from parrots, which covers the 21 species that are bred in Egypt. Feathers are a reliable DNA source, not only for molecular sexing but also for more sophisticated molecular techniques serving to study the parrots populations in the wild [34]. It has been shown previously that DNA isolation protocol is crucial for obtaining reliable PCR and LAMP results. We optimized the DNA isolation protocol and observed that the addition of freshly prepared DTT increases the efficiency of DNA isolation. Our modification was based on the previously published research of Campos and Gilbert [35].

Next, we sought to analyse our samples with a well-established PCR method described by Fridolfsson and Ellegren [13]. This method was repeatedly used with success with a plethora of parrot species, but for *Amazona farinosa* and *Diopsittaca nobilis,* our study is the second to test and confirm that this PCR method is applicable in the aforementioned parrots’ species [14].

Positive results from PCR allowed us to test the feasibility of LAMP application in parrot sex identification. For this task, we selected primers published by Centeno-Cuadros [17]. As primers PSI-W, UCE and CIC-Z were not previously tested for 17 out of 21 species in our collection, we optimized temperatures and incubation time. Our results showed that incubation temperatures can vary from 57 °C to 63 °C. This is in agreement with the literature. The LAMP optimal reaction temperatures span a wide range, as reported previously for LAMP in bird sexing, bacteria or parasite detection [17,36,37]. Primers PSI-W were designed specifically to determine the female sex and they performed well with our sample collection. This was not the case for primer set UCE, which was designed to detect parrot DNA and, in combination with a negative result from PSI-W, should be a determinant of the male sex. As there is only one study that tested these primers previously, our results indicate that new universal primers are needed to confirm male sex in parrots [17]. During the search for other solutions for the detection of parrot DNA, we decided to use CIC-Z primers, which were previously used to differentiate between females and males in the family *Ciconiidae* [24]. Although these primers were originally designed for *Ciconiidae*, they target the CHD gene region, which is relatively conserved across *Neoaves.* These primers showed improved performance in comparison to UCE primers. Our study is the first report to provide information about the detection of males for *Psittaculidae*, *Cacatuidae*, *Psittacidae* families and proved that the use of PSI-W primers in combination with CIC-Z allows a distinction to be made between males and females in six species belonging to these families.

When we compared results from PCR and LAMP, we noticed that seven samples determined as female in LAMP gave no results in PCR. This might be because LAMP is more robust and less sensitive to impurities in isolated DNA that are common when the source of genetic material is feather plucks [38]. Both PCR and LAMP did not work well in samples from three parrot species: *Amazona aestiva*, *Amazona farinosa* and *Nymphicus hollandicus*. This indicates that these species have higher target sequence variation in comparison to other parrot species, but to validate this conclusion, the sequences of target genes have to be obtained and analysed. Knowing these sequences should allow the introduction of the third pair of primers, called loop forward (LF) and loop backward (LB), to accelerate LAMP reaction [39].

## 5. Conclusions

Taken together, this study aimed to test the performance of loop-mediated isothermal amplification (LAMP) in the sex identification of parrots bred in Egypt. We were able to establish the DNA isolation protocol and find optimal conditions for PCR and LAMP. Moreover, we found that further improvements are required in universal LAMP primers for the detection of parrot DNA. We show that the LAMP method is consistent with the established PCR test and in some cases may be more sensitive for female sex identification than the reference PCR test. Furthermore, LAMP potentially allows for an easy amplification confirmation without the use of any additional specialized equipment. This makes it an ideal candidate for field molecular testing in poorly resourced laboratories. Therefore, we conclude that LAMP is a suitable method for routine diagnostic sex identification in parrots.

## Figures and Tables

**Table 1:** Feather samples collected in this study.

Family	Genus	Species	Common Name	Status by IUCN 2019	Number of Birds
Psittacidae	*Ara*	*Ara ararauna*	Blue-and-yellow Macaw	Near Threatened	5
		*Ara chloropterus*	Red-and-green Macaw	Least Concern	8
		*Ara militaris*	Military Macaw	Vulnerable	2
	*Amazona*	*Amazona aestiva*	Blue-fronted Amazon	Least Concern	5
		*Amazona amazonica*	Orange-winged Amazon	---------	5
		*Amazona farinosa*	Southern mealy Amazon	Near Threatened	3
	*Aratinga*	*Aratinga solstitialis*	Sun Parakeet (Sun conure)	Endangered	5
	*Diopsittaca*	*Diopsittaca nobilis*	Northern Red-shouldered Macaw	Least Concern	8
	*Lorius*	*Lorius lory*	Lory	---------	10
	*Myiopsitta*	*Myiopsitta monachus*	Monk Parakeet	Least Concern	12
		*Poicephalus meyeri*	Meyer’s Parrot (red eyes)	Least Concern	4
		*Poicephalus senegalus*	Senegal Parrot	Least Concern	10
	*Psittacus*	*Psittacus erithacus timneh*	Timneh Parrot	Endangered	4
		*Psittacus erithacus*	African Grey Parrot	Endangered	4
	*Pyrrhura*	*Pyrrhura molinae*	Green-cheeked Parakeet	Least Concern	11
Cacatuidae	*Nymphicus*	*Nymphicus hollandicus*	Cockatiel	Least Concern	12
	*Cacatua*	*Cacatua sulphurea*	Yellow-crested Cockatoo	Critically Endangered	5
Psittaculidae	*Melopsittacus*	*Melopsittacus undulatus*	Budgerigar	Least Concern	10
	*Agaprins*	*Agapornis fischeri*	Fischer’s lovebird	Least Concern	15
	*Psittacula*	*Psittacula eupatria*	Alexandrine Parakeet	Near Threatened	4
		*Psittacula krameri*	Rose-ringed Parakeet	Least Concern	8

**Table 2:** The list of primers used in this study.

Primer Set	Primer Name	Oligonucleotide Sequence (5′→3′)	Source
PCR	2550F	GTTACTGATTCGTCTACGAGA	[13]
	2718R	ATTGAAATGATCCAGTGCTTG	
UCE	UCE-F3	GGGAAACAAGGATAAAATTACTCC	[24]
	UCE-B3	TGCCCAGAAAATTCCATTC	
	UCE-FIP	CGAGTGTGTTAAGCACAGTTTTATTTTTTATGGTTAATGA CCTATAGTATCTCC	
	UCE-BIP	GAGGACTGTTCTGCAGGGTATTTTTTTGCTATCTGATTCGAAAAGTC	
PSI-W	PSI-W-F3	CAGTTTCCCTTTCAGGTAAG	[24]
	PSI-W-B3	TCAGTTGCCAAAACAATGG	
	PSI-W-FIP	TTCTTCACAAAGGACACTTTTCTTTTTGTAGTAGCCAAGAAGCCTT	
	PSI-W-BIP	AGGAAAAGACTGGCAATTACTATATGCTAATTTTGGGGAGATAAGATTAATGTAACA	
CIC-Z	CIC-Z-F3	TCACAGAAGATGGAGATTCC	[24]
	CIC-Z-B3	CAACAGAGTTCTGATTTTCTCA	
	CIC-Z-FIP	GCCAAGAAGCTTTGGTCTTGAACTTTTCTCTGGACAACTTGTTCAGT	
	CIC-Z-BIP	ACTACCACCAAGATTCATACCTGATTTTCAGATGGTGAGGATGCTG	

F3 = forward external primer; B3 = backward external primer; FIP = forward internal primer composed by F1c and F2 primers connected by TTTT; BIP = backward internal primer composed by B1c and B2 primers connected by TTTT.

**Table 3:** Conditions used for PCR and LAMP in different parrot species.

Family	Species	Common Name	Positive Control UCE	Sexing		PCR Optimal Annealing Temperature
				PSI-W	CIC-Z	
Psittacidae	*Ara ararauna*	Blue-and-yellow Macaw	---	57 °C/1 h	---	56 °C
	*Ara chloropterus*	Red-and-green Macaw	---	57 °C/1 h	63 °C/1 h	56 °C
	*Ara militaris*	Military Macaw	---	57 °C/1 h	---	56 °C
	*Amazona aestiva*	Blue-fronted Amazon	---	63 °C/1 h	---	50 °C
	*Amazona amazonica*	Orange-winged Amazon	57 °C/1 h	63 °C/1 h	63 °C/1 h	56 °C
	*Amazona farinosa*	Southern mealy Amazon	---	63 °C/1 h	---	56 °C
	*Aratinga solstitialis*	Sun Parakeet (sun conure)	57 °C/1 h	60 °C/1 h	---	52 °C
	*Diopsittaca nobilis*	Northern Red-shouldered Macaw	---	63 °C/1 h	---	52 °C
	*Lorius lory*	Lory	57 °C/1 h	63 °C/1 h	63 °C/1 h	52 °C
	*Myiopsitta monachus*	Monk Parakeet	---	57 °C/1 h	63 °C/1 h	52 °C
	*Poicephalus meyeri*	Meyer’s Parrot (red eyes)	---	57 °C/1 h	63 °C/1 h	56 °C
	*Poicephalus senegalus*	Senegal Parrot	---	63 °C/1 h	63 °C/1 h	56 °C
	*Psittacus erithacus*	African Grey Parrot	---	60 °C/1 h	63 °C/1 h	50 °C
	*Psittacus erithacus timneh*	Timneh Parrot	---	57 °C/1 h	---	50 °C
	*Pyrrhura molinae*	Green-cheeked Parakeet	---	57 °C/1 h	63 °C/1 h	52 °C
Cacatuidae	*Nymphicus hollandicus*	Cockatiel	---	57 °C/1 h	---	50 °C
	*Cacatua sulphurea*	Yellow-crested Cockatoo	---	57 °C/1 h	63 °C/1 h	56 °C
Psittaculidae	*Melopsittacus undulatus*	Budgerigar	---	63 °C/1 h	63 °C/1 h	52 °C
	*Agapornis fischeri*	Fischer’s lovebird	57 °C/1 h	57 °C/1 h	63 °C/1 h	52 °C
	*Psittacula eupatria*	Alexandrine Parakeet	---	60 °C/1 h	---	56 °C
	*Psittacula krameri*	Rose-ringed Parakeet	57 °C/1 h	57 °C/1 h	---	56 °C

—Optimal conditions not determined.

## Data Availability

No data was archived or deposited for this work.
